# Bed Sensor Technology for Objective Sleep Monitoring Within the Clinical Rehabilitation Setting: Observational Feasibility Study

**DOI:** 10.2196/24339

**Published:** 2021-02-08

**Authors:** Maartje M S Hendriks, Jaap H van Lotringen, Marije Vos-van der Hulst, Noël L W Keijsers

**Affiliations:** 1 Department of Research Sint Maartenskliniek Nijmegen Netherlands; 2 Department of Rehabilitation Donders Institute for Brain, Cognition and Behaviour Radboud University Medical Center Nijmegen Netherlands; 3 Department of Rehabilitation Sint Maartenskliniek Nijmegen Netherlands

**Keywords:** continuous sleep monitoring device, bed sensor technology, mHealth, nocturnal heart rate, nocturnal respiratory rate, nocturnal movement activity, neurological disorders, incomplete spinal cord injury, stroke, inpatient rehabilitation, clinical application

## Abstract

**Background:**

Since adequate sleep is essential for optimal inpatient rehabilitation, there is an increased interest in sleep assessment. Unobtrusive, contactless, portable bed sensors show great potential for objective sleep analysis.

**Objective:**

The aim of this study was to investigate the feasibility of a bed sensor for continuous sleep monitoring overnight in a clinical rehabilitation center.

**Methods:**

Patients with incomplete spinal cord injury (iSCI) or stroke were monitored overnight for a 1-week period during their in-hospital rehabilitation using the Emfit QS bed sensor. Feasibility was examined based on missing measurement nights, coverage percentages, and missing periods of heart rate (HR) and respiratory rate (RR). Furthermore, descriptive data of sleep-related parameters (nocturnal HR, RR, movement activity, and bed exits) were reported.

**Results:**

In total, 24 participants (12 iSCI, 12 stroke) were measured. Of the 132 nights, 5 (3.8%) missed sensor data due to Wi-Fi (2), slipping away (1), or unknown (2) errors. Coverage percentages of HR and RR were 97% and 93% for iSCI and 99% and 97% for stroke participants. Two-thirds of the missing HR and RR periods had a short duration of ≤120 seconds. Patients with an iSCI had an average nocturnal HR of 72 (SD 13) beats per minute (bpm), RR of 16 (SD 3) cycles per minute (cpm), and movement activity of 239 (SD 116) activity points, and had 86 reported and 84 recorded bed exits. Patients with a stroke had an average nocturnal HR of 61 (SD 8) bpm, RR of 15 (SD 1) cpm, and movement activity of 136 (SD 49) activity points, and 42 reported and 57 recorded bed exits. Patients with an iSCI had significantly higher nocturnal HR (*t*_18_=−2.1, *P*=.04) and movement activity (*t*_18_=−1.2, *P*=.02) compared to stroke patients. Furthermore, there was a difference between self-reported and recorded bed exits per night in 26% and 38% of the nights for iSCI and stroke patients, respectively.

**Conclusions:**

It is feasible to implement the bed sensor for continuous sleep monitoring in the clinical rehabilitation setting. This study provides a good foundation for further bed sensor development addressing sleep types and sleep disorders to optimize care for rehabilitants.

## Introduction

An important aim of rehabilitation therapy for patients with neurological disorders such as stroke or spinal cord injury (SCI; including incomplete SCI [iSCI]) is to develop skills needed for independent living. Although rehabilitation focuses mainly on activities during the day, sleep is also important for rehabilitation. Patients with a stroke or SCI can face sleep disturbances including sleep disorders [1-3]. Up to 60% of patients with stroke or SCI suffer from sleep disorders [1,2], which is high compared to the 24% of people with sleep problems in the general European population [4]. Those sleep disturbances may interfere with their long-term rehabilitation process for a variety of reasons in terms of lowering motivation, energy, and concentration, which are needed to fully participate in the rehabilitation process [3,5]. Sufficient and adequate sleep promotes rehabilitation by gaining physical and psychological energy [3,6]. It also improves implicit learning of motor skills [7] and cognitive performance [8-10], which may result in performance improvements [11]. Furthermore, sleep positively affects patients’ daily activity and reduces the risk of diabetes, cardiovascular diseases, and development of metabolic disorders [1,2,8,10,12,13], which in its totality contributes to the quality of life. Hence, sleep is essential for optimal inpatient rehabilitation and long-term health, indicating the importance of sleep assessment.

At the moment, polysomnography (PSG) is considered one of the most comprehensive methods for sleep assessment and therefore the gold standard for the diagnosis of many sleep disorders [14,15]. Unfortunately, PSG is generally experienced as impractical, expensive, and limited in accessibility during inpatient rehabilitation in specialized rehabilitation centers [3,14,16-18]. Subjective measurements, such as self-report questionnaires, are inexpensive and easy to implement [16]. However, they summarize only the perception of the patient and are prone to missing data [19,20]. Therefore, a wide range of sleep technology devices used to assess sleep have been developed [18,21,22]. The main aim of those devices is to be less expensive, less invasive, and more accessible for assessing sleep compared to PSG. These sleep technology devices can be classified into contact (actigraphy, electroencephalography) and contactless (ballistocardiography [BCG], microphone, infrared, video camera, echo-based, or mobile) devices [19,22]. The accuracy and reliability of these devices have been continuously increased with the advances of technology [19]; however, most of them have several disadvantages when applied in a clinical inpatient rehabilitation setting. Disadvantages of contact devices comprise discomfort and disruption of sleep, potential for misplacement, and limited data storage and battery capacity [17,18,22]. Contactless portable devices based on infrared, video camera, echo, or mobile phones have the disadvantages that they might lead to incorrect measurements when there are multiple patients in one room, patients change rooms, or nursing staff visits the patient during the night [17,18,22]. Furthermore, most do not monitor heart rate (HR) and respiratory rate (RR), two vital signs during inpatient rehabilitation [23]. A solution to overcome the aforementioned disadvantages of various sleep measuring methods can possibly be found in the recent development of unobtrusive, contactless, portable sensor–based devices for objective sleep analysis based on BCG. BCG technology consists of a highly sensitive pressure sensor, which can measure HR, RR, and movement activity (body movements), which are parameters used in sleep monitoring [24,25]. Studies have reported nocturnal HR and RR coverage ranging between 83% and 92% [26,27]. In this way, a portable bed sensor based on BCG can measure multiple nights without causing any discomfort over a longer period of time. Furthermore, this bed sensor is not prone to disturbance of its signal by others in comparison to the other contactless devices. Therefore, the portable bed sensor based on BCG has potential to be suitable for sleep monitoring in long stay rehabilitation inpatients such as stroke or SCI [28-30].

To our knowledge, portable bed sensors have not yet been implemented during inpatient rehabilitation, although they could be useful in clinical practice. Only a few studies have included a small number of patients using portable bed sensors under research circumstances in a hospital setting [31-33]. However, human, technological, or environmental issues might come along with implementation of sensors in noncontrolled settings [34-36]. Therefore, the aim of this study was to investigate the feasibility of an unobtrusive, contactless, portable bed sensor for continuous sleep monitoring overnight in a clinical rehabilitation SCI and stroke ward. Feasibility was examined based on missing measurement nights. To examine the ability of the bed sensor to capture sleep-related parameters, the following secondary outcomes were analyzed: coverage percentages of HR and RR, missing HR and RR periods, interruptions of HR and RR signals due to bed exits, and the discrepancy in number of reported and recorded bed exits. Additionally, descriptive data on average nocturnal HR, RR, and movement activity for iSCI and stroke patients were reported. We hypothesized that the bed sensor is feasible in inpatient rehabilitation and is considered feasible if at least 95% of the nights were captured and the total coverage of nocturnal HR and RR was above 80% [26,27,36]. Based on the literature, a higher nocturnal HR for iSCI compared to stroke patients was hypothesized [37,38], which serves as a first indication of group validity of the bed sensor.

## Methods

### Study Design

This observational cohort study was carried out at the Sint Maartenskliniek (Ubbergen, the Netherlands). This study was part of an overarching study [39], which aimed to develop a sensor-based technological platform to monitor gait and sleep. It was performed in the clinical treatment environment of the rehabilitation department to integrate the intervention in a realistic setting. Interaction between participant and researcher was kept to a minimum to prevent interference with clinical practice. The study was conducted in accordance with the principles of the Declaration of Helsinki (64th World Medical Association General Assembly, Fortaleza, Brazil, October 2013) and approved by the Medical Ethical Research Committee of Arnhem-Nijmegen (4222-2018).

### Participants

Twelve iSCI patients (ASIA [American Spinal Injury Association] scale C/D) and 12 first-ever stroke survivors (Functional Ambulation Category score≥2) were recruited (both sexes, age≥16 years), as they both have relatively long inpatient hospital stay durations. The following criteria had been applied in relation to the overarching project: minimum age of 16 years, not wheelchair-bound, participation in ambulation therapy, no other comorbidities affecting patients’ ambulatory function, and no use of an anti-decubitus air bed mattress because of interference with the measurement. Participants who were unable to grant permission to participate in the study due to language issues or cognitive impairment were excluded. 

Patients who were already admitted to the inpatient clinical rehabilitation ward of the Sint Maartenskliniek were pre-assessed for eligibility for study participation. The Montreal Cognitive Assessment test score, which was already performed by the responsible rehabilitation physician for usual care, was used to determine if the patient was capable of participating in the study. Eligible patients were asked to voluntarily participate in the study, and participation was not incentivized. After patients were asked to participate, they had time to consider participation before deciding whether or not to participate in the study. There was no intrusion on the patient’s rehabilitation process, and patients could follow their normal care program based on their personal needs. Patients were hospitalized for both day and night during weekdays (sometimes during weekends) for several weeks. Written informed consent was obtained from all participants before the start of the measurements.

### Instrumentation

A portable bed sensor, the Emfit QS sleep tracker (Emfit Ltd; 542 mm × 70 mm × 1.4 mm), was used. The sensor consists of thin elastic lightweight polymer layers separated by air voids and coated with electrically conductive, permanently polarized layers. The Emfit bed sensor was placed under the bed mattress at the thoracic area of the sleeping patient [40]. Changes in pressure distribution generate a charge on the electrically conductive surfaces of the sensor, which can be measured as a current or a voltage signal [32]. The sensor was compatible with IEEE 802.11b/g/n networks, an international standard local area network protocol, and provides data about HR, RR, and movement activity every 4 seconds. Movement activity was expressed by activity points (AP) in which a higher number of AP corresponds with larger body movements. Small movements with the arm will result in fewer AP compared to whole body movements if sleep position changes. Bed exits were identified by the sensor if no pressure was detected. Bed exits were described by duration and frequency. The Emfit bed sensor needs at least one hour of recording before generating a data file. Data were sent to the Emfit server [41].

The Emfit bed sensor was designed for people who lie in bed with the intention to sleep. As the Emfit bed sensor has not been validated to indicate the moment of sleep onset and offset in iSCI and stroke patients, a sleep questionnaire was filled out manually by the patient himself or herself (or with help of the nursing staff) at the end of every night regarding bedtime, time of awakening, bed exits, and use of sleep medication. Those questions were based on the Pittsburgh Sleep Quality Index referring to “last night” instead of “the last month” [42].

### Procedure

After written informed consent, the bed sensor was placed under the bed mattress of the participant by the researcher, and the sleep questionnaires were handed over to the participant. The bed sensor remained under the bed mattress for 1 week. During the weekend, most patients were allowed to stay at home for one or two nights for rehabilitation purposes. If a participant was transferred to a different room, the nurse was responsible for moving the bed sensor. If there were any errors or questions regarding the bed sensor during the measurement week, the researcher visited the patient. Visits by the researcher during the measurement week were considered as interference and registered. At the end of the measurement week, the researcher collected the bed sensor and the questionnaires. Only one researcher (MMSH) was involved in the described procedure and was not blinded to the study outcomes.

### Outcome Measures

The primary outcome for feasibility was the percentage of missing measurement nights. A night was considered as missing if data were not available. Furthermore, reasons for missing nights were noted. To examine the ability of the bed sensor to capture sleep-related parameters, the following secondary outcomes were analyzed: coverage percentages of HR and RR, distribution of missing HR and RR periods based on duration, interruptions of HR and RR signals due to bed exits, and the discrepancy in number of reported and recorded bed exits. The coverage percentages are the percentages of timestamps in which HR or RR data were detected by the bed sensor. Because duration of missing data might have clinical impact, the missing HR and RR data periods per night were presented in time categories of <31, 31-60, 61-120, and >120 seconds. Furthermore, the sensor registers a bed exit if no pressure is recorded. As a result of getting in or out bed, HR and RR signals could be interrupted prior to or after a bed exit while the sensor measures pressure. These adjoining time intervals of missing HR and RR signals were calculated and referred to as response time. This response time of HR and RR due to bed exits was not included in previous outcomes but was investigated separately. The discrepancy between the number of bed exits reported by the questionnaire and the number of bed exits recorded by the bed sensor was described. Average nocturnal HR was calculated for each night as beats per minute (bpm), RR as cycles per minute (cpm), and movement activity as AP. Interference by the researcher if a participant or nurse noticed and reported an error was noted. In addition, the number of patients who had to switch rooms and the complaints regarding the usage of the bed sensor were obtained.

### Data and Statistical Analysis

The Emfit company provided bed sensor data in CSV files on HR, RR, movement activity, and bed exits, based on their algorithm. A custom MATLAB (R2017b, Version 9.3.0.713579, The MathWorks Inc) script was used for processing and analyzing the sleep period data regarding HR, RR, and movement activity recorded by the bed sensor. For analysis of secondary outcomes, measurement nights were only included if bed sensor data and a complete filled out sleep questionnaire corresponding to that night were available. The questionnaire was used to determine the sleep period, time points of sleep onset, and moments of waking up during each night. Missing data periods due to bed exits were excluded from the coverage percentage and missing data calculations. Statistical analyses were performed with SPSS (version 20.0; IBM Corp) and RStudio (version 1.2.5042; RStudio, PBC). The level of statistical significance was set at *P*<.05. Data were tested for normality using the Shapiro-Wilk test. A *t* test was used for normally distributed data, and a Wilcoxon signed rank test (paired) or Mann-Whitney *U* test was used for nonnormally distributed data. For categorical data, a chi-square test was used. Descriptive statistics were presented as mean (SD) if data were normally distributed and as median (range, minimum-maximum) otherwise. The bed sensor was considered feasible if at least 95% of the nights were covered and the total coverage percentage of nocturnal HR and RR was above 80% [36]. All outcome measures were reported for the in-hospital iSCI and stroke patients separately.

## Results

In total, 25 participants (12 iSCI, 13 stroke) were included in this study. One stroke participant dropped out on the first day because of the experienced additional cognitive load to participate in the study. Therefore, data of 24 participants were analyzed. No statistical differences in sex, age, premorbid sleep problems, or the use of sleep medication were found between the iSCI and stroke groups (Table 1).

**Table 1:** Characteristics of participants.

Characteristic		iSCI^a^	Stroke	*P* value	Chi-square (*df*)
**Demographics**					
	Participants, n	12	12	N/A^b^	N/A
	Gender (male/female), n	9/3	7/5	.39	0.8 (1)
	Age (years), mean (SD)	63.4 (12.9)	68.7 (9.0)	.22	N/A
	Days since injury, mean (SD)	83.8 (67.4)	47.0 (25.6)	.09	N/A
	Premorbid sleep problems, n	0	0	>.99	0 (1)
	Sleep medication, n	2	2	>.99	0 (1)
**iSCI characteristics**					
	ASIA^c^ (AIS^d^ C/D), n	1/11	N/A	N/A	N/A
	SCI level (>T6/<T6), n	7/5	N/A	N/A	N/A
	Lesion level (cervical/thoracic/lumbar), n	4/6/2	N/A	N/A	N/A
**Stroke characteristics**					
	Stroke location (left/right), n	N/A	7/5	N/A	N/A
	Stroke category (ischemic/hemorrhagic), n	N/A	10/2	N/A	N/A
	Stroke type (cortical/subcortical/lacunar), n	N/A	9/1/2	N/A	N/A

^a^iSCI: incomplete spinal cord injury.

^b^N/A: not applicable.

^c^ASIA: American Spinal Injury Association.

^d^AIS: ASIA Impairment Scale.

A total of 67 nights with a median of 5 (range 3-7) per person for iSCI patients and 65 nights with a median of 5.5 (range 5-7) per person for stroke patients were measured, adding up to a total of 132 measurement nights. Of the 132 intended measurement nights, 5 (3.8%) had missing sensor data (3 iSCI, 2 stroke). The reasons for missing sensor data nights were errors with the Wi-Fi connection (2), the sensor slipping away from the bed mattress (1), and unknown reasons (2). Those errors did not interfere with the sleep of the participants, and the participants did not report any hindrance from the bed sensor. Twenty-seven nights were excluded (20.5%) due to incomplete questionnaires making it impossible to detect the sleep period (7 iSCI, 20 stroke). One night (0.8%) of a stroke patient could not be analyzed due to inconsistency regarding end of bedtime between the sensor data and questionnaire. Therefore, a total of 99 (57 iSCI, 42 stroke) measurement nights of 20 patients (11 iSCI, 9 stroke) could be analyzed. The average number of hours of indicated sleep per night was 8 hours and 1 minute (SD 1 hour and 7 minutes) for the iSCI group and 8 hours and 10 minutes (SD 33 minutes) for the stroke group (*t*_18_=0.3, *P*=.74).

Table 2 shows the coverage percentages, mean numbers of missing data periods per time category, and durations of HR and RR signal interruptions due to bed exits. For the iSCI group, coverage percentages of 97% for HR and 93% for RR were found. Stroke participants had coverage percentages of 99% and 97% for HR and RR, respectively. Two-thirds of the missing HR and RR periods had a short duration of less than 121 seconds. The average duration of bed exits per night was 342 (SD 168) seconds for the iSCI group and 257 (SD 121) seconds for the stroke group (*t*_16_=−1.2, *P*=.23). The HR response time (66 seconds; range 0-3972 seconds) to bed exits was significantly shorter compared to the RR response time (163 seconds; range 0-3226 seconds) (*z*=−9.0, *P*<.001). Response time before bed exits (0 seconds; range 0-3226 seconds) was significantly shorter than the response time after bed exits (99 seconds; range 0-3972 seconds) (*z*=−8.4, *P*<.001).

**Table 2:** Coverage percentages, mean number of missing data periods per time category (<31, 31-60, 61-120, and >120 s), and response time due to bed exits of nocturnal HR and RR for iSCI and stroke patients recorded by the bed sensor.

Group		Coverage (%), mean (SD)	Missing data periods per night, mean (SD)				Response time due to bed exits (s), mean (SD)	
			<31 s	31-60 s	61-120 s	>120 s	Before	After
**iSCI^a^**								
	HR^b^	96.8 (4.5)	3.1 (2.8)	1.8 (2.1)	1.5 (1.6)	1.4 (2.0)	9.7 (7.5)	141.1 (261.1)
	RR^c^	92.5 (4.7)	4.6 (1.9)	2.1 (1.0)	2.3 (0.9)	4.6 (2.4)	87.1 (100.1)	240.8 (294.7)
**Stroke**								
	HR	99.3 (0.6)	2.0 (1.2)	0.8 (0.8)	0.7 (0.5)	0.5 (0.7)	8.4 (8.6)	36.2 (25.3)
	RR	96.7 (2.6)	3.1 (1.7)	2.0 (1.5)	1.6 (1.6)	2.4 (2.0)	61.8 (78.9)	103.1 (46.5)

^a^iSCI: incomplete spinal cord injury.

^b^HR: heart rate.

^c^RR: respiratory rate.

Patients with iSCI reported a total of 86 bed exits by questionnaire, of which 84 were recorded by the bed sensor. Stroke patients reported a total of 42 bed exits, whereas 57 bed exits were recorded. There was a difference between self-reported and recorded bed exits per night in 15 of the 57 nights (26%) for iSCI and in 16 of the 42 nights (38%) for stroke patients (Table 3).

**Table 3:** Difference in reported and recorded bed exits per night for iSCI and stroke patients.

Group	Frequency Δ^a^ of reported (questionnaire) and recorded (sensor) bed exits								
	−4	−3	−2	−1	0	+1	+2	+3	+4
iSCI^b^	0	0	1	5	42	9	0	0	0
Stroke	0	0	5	8	26	3	0	0	0

^a^Negative value indicates fewer reported bed exits; positive value indicates more reported bed exits.

^b^iSCI: incomplete spinal cord injury.

The nocturnal HR in iSCI (72 bpm, SD 13 bpm) was significantly higher compared to the stroke group (61 bpm, SD 8 bpm; *t*_18_=−2.1, *P*=.04). Nocturnal RR was 16 (SD 3) cpm and 15 (SD 1) cpm for iSCI and stroke, respectively (*t*_18_=−2.4, *P*=.21). Average nocturnal movement activity was significantly different between iSCI (239 AP, SD 116 AP) and stroke (136 AP, 49 AP) (*t*_18_=−1.2, *P*=.02). The median percentage for movement activity of large movements (>500 AP) was significantly larger in iSCI patients (5.7%, range 1.8%-28.2%) compared to stroke patients (2.8%, range 0.4%-4.9%) (*z*=2.2, *P*=.03). Furthermore, no errors were noted, no participants had to switch rooms, and none of the participants had any complaints regarding the usage of the bed sensor.

## Discussion

The aim of this study was to investigate the feasibility of an unobtrusive, contactless, portable bed sensor for continuous sleep monitoring overnight in a clinical rehabilitation SCI and stroke ward. Sensor data were available for more than 95% of the measured nights. The bed sensor was able to capture HR and RR with high coverage percentages between 92% and 99%, with only short missing HR and RR periods during the night and some interruptions of the HR and RR signals caused by bed exits. iSCI patients had significantly higher nocturnal HR and movement activity than stroke patients. Moreover, no complaints were mentioned regarding the use of the bed sensor. These findings indicate the feasibility of an unobtrusive, contactless, portable bed sensor for continuous sleep monitoring overnight within a clinical setting.

The 3.8% (5/132) missing measurement nights fell within the 5% boundary for feasibility and are in line with literature regarding missing data of various sensor technologies [36,43,44]. Another important positive finding of this study was the high coverage rates (>92%) of HR and RR in both patient groups. These coverage percentages largely exceeded the set boundary of 80% and are at the upper limit of what has been found in literature; studies found average coverage percentages of 83% [26] and 93% [27] for HR and 87% [26] for RR using unobtrusive, contactless, portable sensors under bed mattresses at home and in sleep centers. A possible explanation for our slightly higher coverage percentages might be the exclusion of bed exits and response time due to bed exits. However, patients are not asleep around bed exits, which causes those periods to be clinically less relevant for HR and RR signals. Furthermore, the missing data due to bed exits were mostly short periods of signal interruptions (<31 seconds). Including bed exits in the coverage percentages would reduce the coverage by less than 3%: iSCI HR 94.6% (SD 5.0) and RR 89.6% (SD 6.0), stroke HR 97.9% (SD 1.3) and RR 95.0% (SD 3.0)). Therefore, the clinically relevant nocturnal HR and RR signals could almost entirely be monitored with the bed sensor.

A high coverage of HR and RR is essential to assess HR- and RR-based functional sleep outcomes. Possible interesting HR- and RR-based functional outcomes for clinicians from the bed sensor could be (1) time in bed, (2) sleep latency, (3) sleep efficiency, (4) total time awake, (5) total sleep time, (6) sleep stages (%), and (7) apnea-hypopnea index [1,12,32,45,46]. To determine these functional sleep outcomes, it is important that HR and RR, as well as movement, can properly be measured with the bed sensor. So far, the bed sensor has appeared to be suitable for measurement of HR and RR in laboratory studies with healthy subjects [47-49]. Previous studies found significantly higher HR in low paraplegia SCI patients compared to healthy controls [37] and similar HR in stroke patients and healthy controls [38]. The higher HR in iSCI compared to stroke patients in this study supports higher nocturnal HR in iSCI patients. Although nocturnal RR data in SCI and stroke patients in literature is lacking, nocturnal RR was within the range of a normal population [50]. Studies reporting on nocturnal movement activity are scarce. Based on this study, iSCI patients seem to have a significantly higher percentage of large body movements compared to stroke patients. The abovementioned findings support the bed sensor's ability to discriminate between different groups and is a first indication toward group validity. It suggests that HR, RR, and nocturnal movement activity can be monitored by bed sensors on the rehabilitation ward, but this needs further study and comparison with healthy controls.

Despite the wide range of sleep technology devices, the perfect sleep assessment method does not yet exist: all methods have their advantages and disadvantages [14]. Disadvantages of the portable bed sensor are that the sensor cannot be used in combination with an airflow mattress and that it is designed for sleep detection with intention to sleep. However, in-hospital patients are often inactive due to physical impairments and therefore spend a lot of time in their bed without the intention to sleep, which the sensor cannot distinguish from being asleep. Hence, further validation of the portable bed sensor is needed, and attention must be paid to the overestimation of sleep time, sleep onset, and wake/sleep periods in a greater proportion of the rehabilitation center population [20,46,51].

A limitation of our study was that the data analysis was dependent on completely filled out sleep questionnaires. During our study, we were confronted with a disadvantage of using sleep questionnaires that has been reported previously [14,20]. Patients, especially stroke patients with mild cognitive impairment, did not fill them out regularly and accurately, which was supported by the difference in reported and recorded bed exits. As a consequence, a large proportion (27/132, 20.5%) of measurement nights could not be used in data analysis. In contrast, only 3.8% (5) of the nights were missing due to technical errors. Nonetheless, subjective sleep measures assess more habitual patterns of sleep and sleepiness compared to the objective monitoring of different body functions during sleep [14,16]. Therefore, combining subjective and objective measures of sleep may provide a more comprehensive, continuous evaluation of sleep quality. The bed sensor is a more robust way of sleep monitoring and can complement the subjective sleep assessment in the clinical setting. Therefore, future research should study more extensively the validity of the unobtrusive, contactless, portable bed sensor in immobilized patients within rehabilitation populations, in comparison with healthy controls.

In conclusion, the unobtrusive, contactless, portable bed sensor is a promising and feasible instrument to monitor sleep in the clinical rehabilitation setting. This provides a good foundation for further development of these types of sensors targeting sleep types and sleep disorders.

## Abbreviations

AP: activity points
ASIA: American Spinal Injury Association
BCG: ballistocardiography
bpm: beats per minute
cpm: cycles per minute
HR: heart rate
iSCI: incomplete spinal cord injury
PSG: polysomnography
RR: respiratory rate
SCI: spinal cord injury
